# Perioperative splanchnic perfusion variation around colorectal surgery using both indocyanine green spectrophotometry and fluorescence angiography

**DOI:** 10.1007/s00464-026-12680-1

**Published:** 2026-03-02

**Authors:** Paolo Enrico Meneghesso, Alice Moynihan, Ashokkumar Singaravelu, Conan McCaul, Jeffrey Dalli, Ronan A. Cahill

**Affiliations:** 1 UCD Centre for Precision Surgery, University College Dublin, Dublin, Ireland; 2 Department of Biomedical Sciences, Humanitas University, Pieve Emanuele, Milan, Italy; 3 Department of Anaesthesiology, Mater Misericordiae University Hospital, Dublin, Ireland; 4 Department of Surgery, Mater Misericordiae University Hospital, 47 Eccles Street, Dublin 7, Ireland

**Keywords:** Indocyanine green, Splanchnic perfusion, Spectrophotometry, LiMON, Near-infrared laparoscopy, Near-infrared fluorescence angiography

## Abstract

**Background:**

Indocyanine green (ICG) fluorescence enables validated clinical assessment of both global splanchnic perfusion, via specialised peripheral pulse spectrophotometry, and local intestinal perfusion, via near-infrared fluorescence angiography (ICGFA). This exploratory study investigated perioperative variations and correlations in measurements by these technologies, including their possible relevance with outcomes, in patients undergoing colorectal surgery.

**Methods:**

In consenting patients, plasma disappearance rate (PDR) and percentage of residual ICG at 15 min (ICGR15) were measured preoperatively, intraoperatively and on postoperative day four using ICG-pulse spectrophotometry (LiMON, Getinge). ICGFA (Pinpoint, Stryker) was visually judged by an experienced surgeon concurrently with LiMON assessment intraoperatively and with quantitative analysis of fluorescence intensity–time curves in a subgroup of recorded procedures.

**Results:**

Twenty-eight patients were included, of whom 22 (79%) underwent segmental colorectal resection with primary anastomosis in 19 (68%). While no significant within-subject variation in LiMON-derived perfusion indices evidenced across measurement time points, there was substantial inter-subject variation (notably with age > 65 years and distal resections), especially intraoperatively. Patients having distal resections tended towards greater postoperative rebound. Although patients who developed complications exhibited relatively reduced ICGR15 intraoperatively (*p* = 0.041), there was no significant association with severe complications. Exploratory regression analyses found a modest association between spectrophotometric PDR values and fluorescence-derived parameters, including maximum intensity and time to peak intensity.

**Conclusion:**

Intraoperative splanchnic perfusion varies substantially, particularly during distal colorectal resections. Although qualitative ICGFA may not reflect global hypoperfusion, quantitative fluorescence analysis shows concordance with spectrophotometric findings, supporting its potential as a complementary intraoperative perfusion-monitoring tool in colorectal surgery.

**Supplementary Information:**

The online version contains supplementary material available at 10.1007/s00464-026-12680-1.

Adequate splanchnic and intestinal perfusion are fundamental for healing and recovery after colorectal surgery, with impaired perfusion being associated with anastomotic leakage (AL) in patients undergoing restorative resections. While adequate splanchnic perfusion is usually assumed in patients undergoing elective surgery, gut blood flow can vary even in the face of normal systemic blood pressure due to physiological adaptation mechanisms such as “splanchnic shunting” (i.e. redistribution of blood volume from the splanchnic to systemic circulations to maintain central venous return). This reflex can be triggered by anaesthesia and sepsis while pneumoperitoneum during minimally invasive surgery (MIS) can also affect gut perfusion [1, 2]. However, splanchnic perfusion is not routinely measured perioperatively with only surgeon judgements being used intraoperatively and that is generally limited to the gross appearances of the intestinal region of interest.

Validated, commercial technologies based on the pharmacokinetic and fluorescence properties of an approved intravenously administered dye, namely indocyanine green (ICG), now exist to inform surgeons better perioperatively. Specifically peripheral pulse spectrophotometry (PPS, also known as pulse dye densitometry) and ICG fluorescence angiography (ICGFA) indicate general splanchnic and local perfusion quality, respectively. Tuned PPS exploits as its basis ICG’s high (> 70%) hepatic circulatory extraction before its non-metabolised secretion into bile without enterohepatic recirculation [3, 4]. As ICG clearance depends so on both hepatosplanchnic blood flow and hepatocellular function [5–7], in the presence of normal liver function, quantificative measurement of ICG relates hepatosplanchnic perfusion [3, 8–16]. Specialised PPS has been proven to accurately do this in intensive care settings and after liver resection [3, 4]. In contrast, ICGFA allows qualitative, visual judgement of dynamic intestinal tissue perfusion in any segment under inspection by the surgeon viewing the inflow and outflow of administered ICG using a specialised near-infrared equipped endoscope. There is evidence of improved AL rates after both open and MIS colorectal operations when this is used [17–22].

In this exploratory, hypothesis-generating study, we prospectively applied bedside ICG-PPS to assess splanchnic perfusion in patients undergoing colorectal surgery at different perioperative timepoints. Intraoperatively, ICGFA was also performed at the same time as PPS. The primary aim was to describe perioperative perfusion patterns using these technologies and to compare the readings from each device with the other. Secondarily, we explored whether perioperative PPS measurements, alone or in combination with ICGFA kinetics (including in a subgroup those derived postoperatively computationally) might identify patients at increased risk of postoperative complications.

## Methods

In this single-centre, prospective observational cohort study (ClinicalTrials.gov Identifier: NCT04220242) with predefined exploratory analyses (i.e. STROBE checklist-compliant) [23], adult patients undergoing elective colorectal surgery under the same anaesthetic protocol (and anaesthetist) were included on a near-consecutive basis (equipment limitations meant only one patient was possible per scheduled theatre list) between November 2022 and November 2023. Written informed consent was obtained from all participants in accordance with institutional ethics approval (Mater Misericordiae University Hospital, Dublin, Ireland—1/378/2092).

### Clinical cohort

In general, all patients having major colorectal resection in our unit are monitored in a high dependency unit for the first 24 h postoperatively, with discharge to the ward documented thereafter subject to the patient meeting defined recovery milestones. Demographics, disease and perioperative data were obtained prospectively. Postoperative outcomes were also recorded, with complications categorised by Clavien-Dindo (CD) classification [24], grouped as CD ≥ 3 (severe complications) versus CD < 3 (none/minor). To allow analysis by procedure group, operations requiring ligation of the ileocolic vessels (e.g. right hemicolectomy, ileocaecal resection) were classified as “proximal” resections. All right hemicolectomies were performed using a complete mesocolic excision (CME) approach, which represents standard practice at our centre [25]. Procedures requiring ligation of the inferior mesenteric vessels (e.g. left hemicolectomy, anterior resection, abdominoperineal resection) were classified as “distal” resections. Cases without pedicle ligation (e.g. stoma formation/closure, diagnostic laparoscopy) were excluded from this subanalysis. Exploratory subanalysis by complication severity was also performed using the CD classification. Notably for this study, all patient consistently underwent the same anaesthetic protocol.

### ICG measurements

Patients, awake and fasting, underwent baseline splanchnic peripheral ICG measurement on the morning of surgery before induction of anaesthesia (T1). The measurement was repeated intraoperatively during surgery under anaesthesia (T2), and again on the fourth day postoperatively (T3). To do this, ICG-PPS (LiMON, PulsioFlex Monitoring Platform, Pulsion Medical Systems, Getinge) was used by a trained investigator each time. In brief, the device involves placement of its finger probe, similar to a pulse oximeter, during and for fifteen minutes after intravenous administration of ICG (0.25 mg/kg). By this, the device calculates and algorithmically outputs two relevant values, plasma disappearance rate (PDR, %/min) and percentage of residual ICG at fifteen minutes (ICGR15, %). PDR correlates positively with splanchnic perfusion while ICGR15 serves as an inverse indicator [3, 4, 7, 14]. In patients undergoing intestinal resection, intraoperative measurements were taken immediately before intestinal transection after major vessel ligation as standard. In other operations, it was performed by surgeon’s discretion as it made sense for the specific procedure. In laparoscopic procedures, measurements were obtained with the pneumoperitoneum desufflated and the patient in level position. In the event of device read failure, a second ICG dose and measurement were permitted at T1 and T3 but not T2 (to avoid operation prolongation). Intraoperatively, ICGFA imaging was performed using the commercially available Pinpoint Fluorescence Imaging System (Stryker Corp, Kalamazoo, MI, USA), simultaneously with spectrophotometry in all cases and utilising the same ICG dose. This system shows simultaneously white light, NIR and combined overlay views to the left of the screen with the rest of the screen displaying the user’s choice of one of these views, allowing for real-time visual assessment of ICGFA for perfusion sufficiency by an experienced surgeon. If ICGFA revealed inadequate perfusion at the initially planned transection site, the level of transection was adjusted to a better-perfused segment. Post hoc analysis of ICGFA recordings was performed to quantify the time-series signals using MATLAB R2024a (Matlab, Mathworks) in a subgroup of patients. To do this, video frames underwent stabilisation via affine geometric transformation using their division into 10 × 12 grid regions. Four well-perfused regions of interest (ROI) were selected from the ICGFA timeframes by a surgeon expert in ICGFA interpretation for subsequent correlation analysis. Maximum fluorescence intensity (*F*_max_) and time to reach maximum intensity (*T*_max_) were calculated from perfused intestinal grid regions.

### Statistical analysis

Data were prospectively recorded in Microsoft Excel and later analysed in IBM SPSS Statistics for Windows, Version 29.0 (IBM Corp., Armonk, NY, USA). Continuous variables were summarised as median and interquartile range (IQR). Normality of continuous variables was assessed using the Shapiro–Wilk test; subsequent analyses were performed using the parametrically appropriate tests. Within each group, paired comparisons between timepoints (e.g. preoperative vs. intraoperative, intraoperative vs. postoperative and preoperative vs. postoperative) were assessed using the Wilcoxon signed-rank test, while overall changes across the three timepoints were explored with the Friedman test and pairwise testing with Bonferroni’s correction. Inter-group comparisons of absolute differences (Δ) and relative percentage changes (Δ%) in PDR and ICGR15 were performed using the Mann–Whitney *U* test. Associations between categorical variables were assessed using Fisher’s exact test. Associations between perioperative perfusion metrics and resection types as well as postoperative outcomes (examined by stratifying patients according to CD classification) were performed. Subgroup analyses were conducted to examine whether the observed relationships varied according to patient characteristics, including age. Outliers were identified visually from graphical distributions and quantitatively using standardised Z-scores, with values exceeding ± 3 considered extreme and excluded by predefined sensitivity analysis performed to assess robustness of findings [26]. Exploratory linear regression analyses examined relationships between fluorescence intensity–time features and LiMON-derived parameters. Two models were specified, one with ICGR15 (model 1) and one with PDR (model 2) as the dependent variable. A two-sided *p*-value < 0.05 was considered statistically significant.

## Results

### Baseline demographics and clinical features

Twenty-eight patients undergoing different operations were studied (Table 1). The median (IQR) age was 68 (60–75.5) years. Seventeen (60%) were male. Fourteen (50%) patients had an ASA score of 2, 12 (43%) patients were ASA 3 and 2 (7%) patients were ASA 4. Twenty-two (79%) patients underwent segmental colorectal resection, of which nineteen (68%) included anastomosis. Among the latter, the most performed operation was right hemicolectomy (*n* = 8), followed by anterior resection (*n* = 5) and left hemicolectomy (*n* = 4). Five patients underwent either formation (*n* = 2) or closure (*n* = 3) of a stoma and one had diagnostic laparoscopy alone. Twenty-one (75%) patients underwent laparoscopic operation, while seven (25%) patients had either open surgery ab initio or early unplanned conversion. The median (IQR) overall HDU stay was 1 (0–2) day, with a median length of hospital stay of 8 (6.3–11) days. Twelve (43%) patients experienced a postoperative complication, with three (10.7%) developing a severe complication (CD ≥ 3) (Table 1).

**Table 1:** Patient demographics with operative type, access and postoperative course overall

Age in years	Median (IQR)	68 (60–75.5)
Sex	Male/female	17/11
ASA score	ASA 2	14
	ASA 3	12
	ASA 4	2
Diagnosis	Colorectal cancer	25
	Inflammatory bowel disease	3
Postoperative course	HDU stay, median (IQR)	1 (0–2) days
	LOS, median (IQR)	8 (6.3–12) days
	Any complication	12
	Clavien-Dindo ≥ 3	3

*ASA* American Society of Anesthesiologists, *IQR* interquartile range, *LOS* length of hospital stay, *HDU* high dependency unit

### PPS assessment

**Fig. 1:**
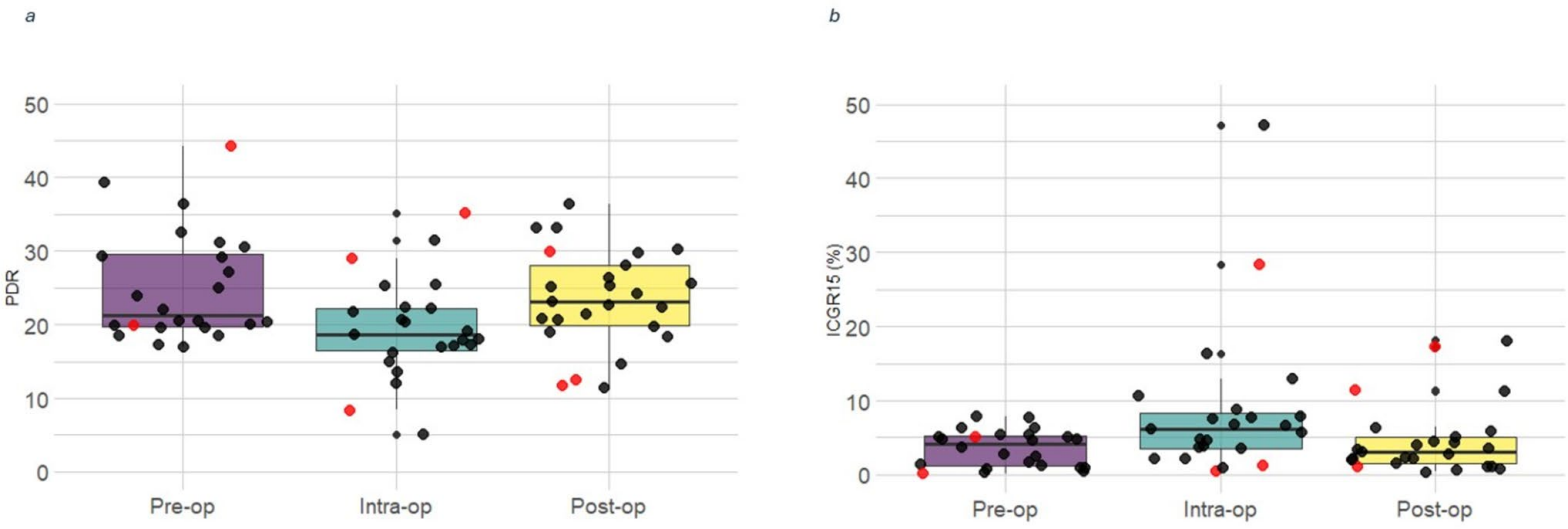
Dot box plots showing median, interquartile range and individual patient values of **a** PDR and **b** ICGR15. Individual patients suffering grade ≥ 3 Clavien-Dindo complications are shown in red

Splanchnic perfusion was assessed at each time point in all patients; however, spectrophotometry data were unavailable for some time points due to inflow detection failures of the device (Table 2). Missing measurements occurred in four patients at T1, five at T2 and three at T3. No patients had more than one missing measurement. Successfully captured spectrophotometry data are shown in Fig. 1 and Table 3. The deviation of several variables from a normal distribution along with limited sample size indicated non-parametric method data analysis for consistency. While variation in PPS metrics was evident between individuals with respect to their response to surgery, no significant within-subject differences in LiMON spectrophotometry values were observed across T1, T2 and T3. Age-related differences were observed intraoperatively (T2) with older patients (≥ 65 years) showing lower PDR and higher ICGR15 values (*p* = 0.039 and *p* = 0.033, respectively). No sex-related differences were detected. Seven (25%) patients exhibited a ≥ 20% reduction in PDR values. In two (7%), the PDR recovered towards baseline at T3 (within ± 20% of preoperative PDR). In three (11%), reductions in PDR persisted postoperatively in whom one experience a CD grade 3 complication. The remaining two patients (7%) had missing readings. Four (14%) patients demonstrated an intraoperative increase > 20% from baseline in PDR. In case, surgical ICGFA judgement was deemed satisfactory.

**Table 2:** Summary of surgical data and clinical outcomes

Operation (*n*)	Subtype (*n*)	Postop complication (%)	Total length of stay, median (IQR)	HDU length of stay, median (IQR)
Colorectal resections with anastomosis (*n* = 19)	Right hemicolectomy (*n* = 8)	5 (63%)	8.5 (8–12)	1 (0.25–2)
	Anterior resection (*n* = 5)	1 (20%)		
	Left hemicolectomy (*n* = 4)	2 (50%)		
	Subtotal colectomy (*n* = 1)	0		
	Ileocaecal resection (*n* = 1)	1 (100%)		
Colorectal resection with end stoma (*n* = 3)	Abdominoperineal resection (*n* = 2)	2 (100%)	8 (7–10.5)	3 (2–3.5)
	Completion proctectomy (*n* = 1)	0		
Other operations (*n* = 6)	Stoma formation (*n* = 2)	0	6 (5–8)	0 (0–0)
	Stoma closure (*n* = 3)	1 (33%)		
	Diagnostic laparoscopy (*n* = 1)	0		

*HDU* high dependency unit

**Table 3:** Overall cohort values of PDR and ICGR15 at each measurement time point

		PDR (%/min)	ICGR15 (%)
Preoperative (T1)		21.3 (19.7–29.6)	4.1 (1.2–5.2)
Intraoperative (T2)		18.7 (16.6–22.2)	6.1 (3.6–8.4)
Postoperative (T3)		23.1 (19.8–28.0)	3.1 (1.5–5.1)
Δ Pre-intra		− 2.6 (− 8.5–1.5)	1.2 (− 1.0–4.5)
	%	4.1% (− 14.9–15.0)	47.8% (− 20.6–345.6)
Δ Pre-post		− 2.1 (− 8.6–0.4)	1.0 (− 0.3–1.7)
	%	− 10.3% (− 31.8–2.0)	36.2% (− 6.3–262.0)
Δ Intra-post		2.8 (− 1.3–7.1)	− 1.7 (− 4.4–0.6)
	%	13.8% (− 6.2–37.7)	− 35.4% (− 64.6–21.3)

Missing values: *n* = 4 preoperatively, *n* = 5 intraoperatively, *n* = 3 postoperatively

Values are reported as medians (IQR)

### Postoperative outcomes analysis

**Fig. 2:**
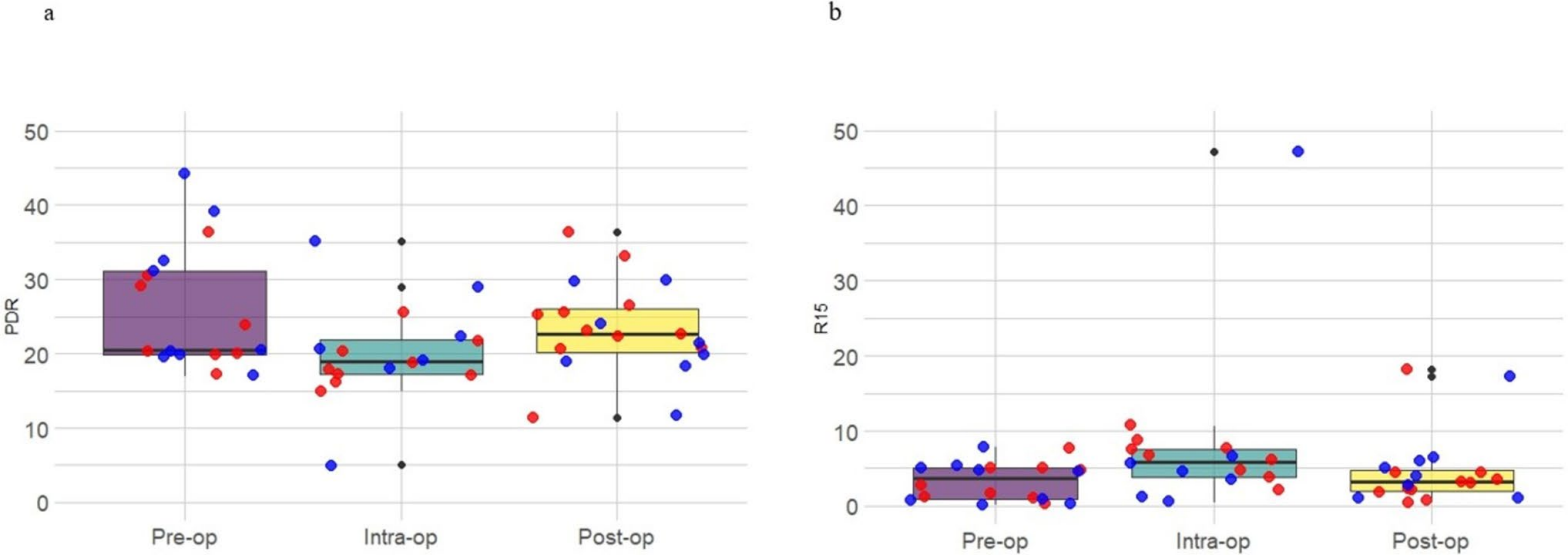
Dot box plots showing the median, interquartile range and individual patient values of **a** PDR and **b** ICGR15. Proximal and distal resections are shown in blue and red dots, respectively

The incidence of postoperative complications did not differ significantly between older (≥ 65 years) and younger patients (< 65 years) (Fisher’s exact test, *p* = 0.705). A significant difference in ΔICGR15 between T2 and T1 measurements (*p* = 0.041) was noted, with patients without complications exhibiting a relatively higher intraoperative increase in ICGR15 compared to those suffering complications (see Supplementary Table 2). However, patients experiencing severe postoperative complications (CD ≥ 3) showed no significant differences in the variability or distribution of PDR and ICGR15 at any time point, nor in any of the Δ or Δ% metrics. Exclusion of one observation with an extreme standardised Z-score ( >|3|) in the sensitivity analysis did not materially alter these results, which remained consistent with the primary analysis (Fig. 2).

### Vascular pedicle analysis (proximal vs. distal)

When stratified by resection type, proximal resections (*n* = 9) showed relatively stable medians of both ICGR15 across the three time points, whereas distal resections exhibited greater descriptive variation in both ICGR15 and PDR (see Table 3). No age-related differences were found between the two groups (Fisher’s exact test, *p* = 0.556). Neither inter-group measurements (Mann–Whitney test) nor intra-group variability (Friedman test) differed significantly at any time point. Across all Δ and Δ% metrics, a trend was observed for the T2–T3 change, suggesting a greater postoperative rebound of the distal resections, although this did not reach statistical significance (*p* = 0.088) (see Supplementary Table 1). The sensitivity analysis, performed by exclusion of the single patient with extreme intraoperative ICGR15 values (*Z*-score >|3|), confirmed this pattern, revealing a significant difference in ΔPDR and ΔICGR15 between T2 and T3 measurements (*p* = 0.012).

### Fluorescence intensity–time analyses

**Fig. 3:**
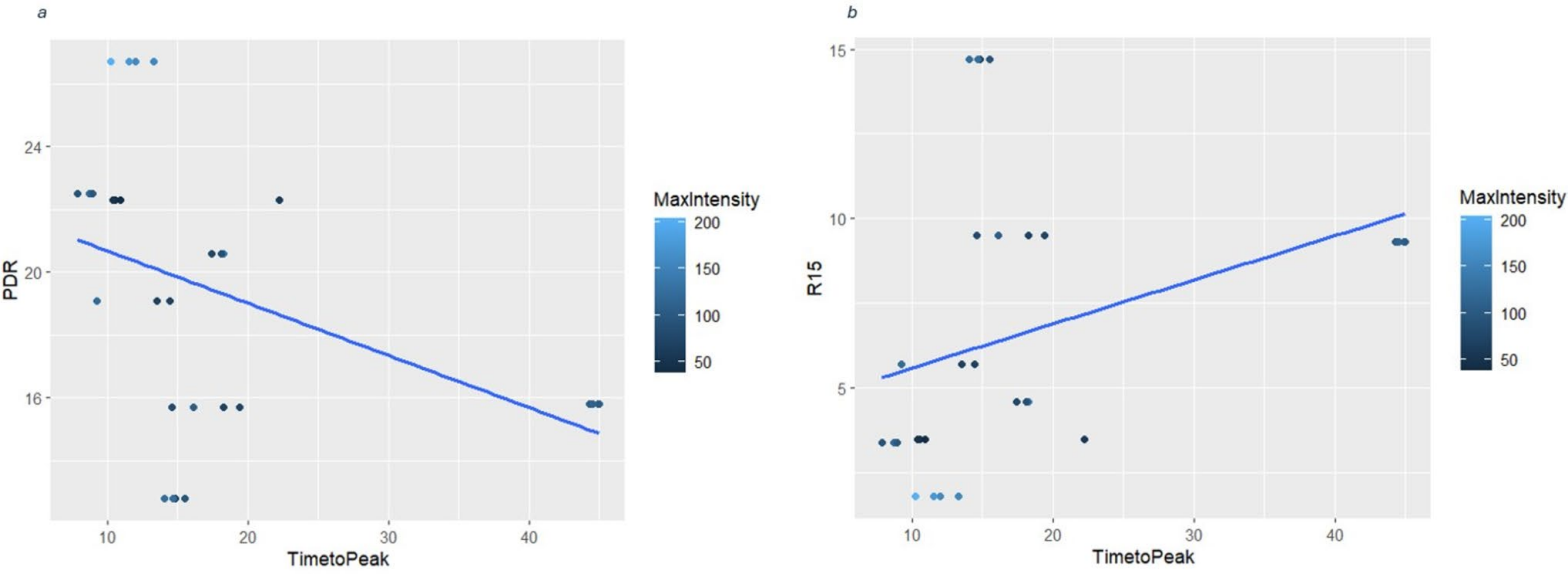
Scatterplots illustrating the relationship between time to maximum fluorescence intensity (T max) and **a** PDR and **b** ICGR15, with fitted regression lines

Fluorescence intensity–time analyses were feasible in eight of the 28 procedures, as corresponding video recordings were available. In exploratory regression analyses, the LGM correlation of ICGFA timeseries with LiMON measurements revealed a multiple R^2^ value of 0.30 (adjusted *R*^2^ 0.25) for model 1 (PDR) and 0.16 (adjusted *R*^2^ 0.10) for model 2 (ICGR15) (Fig. 3 shows the relationship between PDR and ICGR15, respectively, versus time to peak, by maximum intensity and a linear regression line overlaid). Overall, model 1 found statistical significance with an F-statistic of 6.177 (*p* = 0.006), but the effect size is small. Time to reach maximum intensity had a significant negative effect on PDR (estimate − 0.16, *p* = 0.01), while maximum intensity had a significant positive effect (estimate 0.04, *p* = 0.03). Overall, model 2 approached but did not reach statistical significance with an F-statistic of 2.73 (*p* = 0.08). Time to reach maximum intensity had a significant positive effect on ICGR15 (estimate 0.13, *p* = 0.049) while maximum intensity did not show a statistically significant effect (estimate − 0.02, *p* = 0.28).

## Discussion

To our knowledge, this is the first study to apply PPS longitudinally across the perioperative period in elective colorectal surgery and to compare its findings with those obtained from ICGFA. In theory, any impaired splanchnic perfusion occurring intraoperatively (including transiently) may contribute to postoperative morbidity, including ileus or sepsis via relative ischaemia potentially inducing intestinal bacterial translocation [27, 28]. While this study was not appropriately powered to detect significant associations between intraoperative perfusion measures and clinical outcomes, it offers useful methodological insights for future investigations. Understanding splanchnic perfusion patterns perioperatively is also especially relevant in light of the substantial evidence that now exists regarding the use of NIR perfusion angiography during colorectal surgery [18–22]. While assurance of adequate intestinal perfusion, both through the selection of healthy intestinal segments and the avoidance of tension, is an enshrined surgical principle for competent anastomotic construction, this digital adjunct method directly enables recordable visualisation of blood flow [29]. In current practice, surgeons judge ICG perfusion by first seeing the inflow of fluorescence at the region of interest and then judging its briskness (often in comparison with that observed in another area visible within the same field of view) [30]. Global hypoperfusion may undermine this through impaired transection-site perfusion that remains undetected as ICGFA can still appear to show relatively reasonable perfusion to the surgeon [31]. Quantification of ICG signals is increasingly reported in the literature in this field, although it is generally correlated as a single intraoperative time point with postoperative outcome alone [32–34].

In this study, perioperative ICG-derived measures proved feasible and showed observable fluctuations in gut perfusion measurements perioperatively. Such variability here is not uniform but instead subject to considerable inter-individual variation, even when controlling for general anaesthesia, pneumoperitoneum and operative steps that promote splanchnic-systemic shunting. Perfusion characteristics evidenced variation, for example, when age or vascular territory of the operative resection were considered. Although others have reported an age-related decline in ICG clearance or impaired hepatic performance in different cohorts [35, 36], a lack of literature reporting intraoperative assessment of ICG peripheral spectrophotometry in colorectal surgery was noted, thus hampering comparison. No differences were observed in the distribution of PDR and ICGR15 values by site and side-specific patterns became more apparent when dynamic changes (Δ values) between timepoints were considered. In particular, the intraoperative-to-postoperative change in distal resections showed a more pronounced postoperative rebound in perfusion parameters versus a more relative stability seen with proximal resections. This observation warrants further investigation in relation to the well-described differences in anastomotic leak between proximal and distal colorectal procedures, particularly anterior resections [37, 38].

In this limited series, however, observed perfusion patterns were not associated with clinical outcomes. Interestingly, patients experiencing a postoperative course free of any complications showed a significantly higher ΔICGR15 when intraoperative values were compared with the preoperative baseline; however there was no such significance versus patients with severe complications, although numbers in this group were small. Moreover, comparisons performed within each age category (≥ 65 and < 65 years) did not show significant differences in any LiMON measurement between patients with and without complications, suggesting that age may not be a major confounder in these results.

In our exploratory analysis of the recorded surgical videos, both fluorescence-derived parameters (maximum intensity, *F*_max_, and time to reach maximum intensity, *T*_max_) were significantly associated with the PDR of the ICG. Given the limited sample size, these analyses were intended to be hypothesis-generating and are interpreted with caution. Specifically, the higher maximum intensity of the fluorescence in the bowel segment chosen for the transection was positively associated with PDR. Also, a shorter time to reach the maximum fluorescence intensity was associated with higher PDR. A plausible explanation of these findings is that a higher splanchnic perfusion would be expected to accelerate the hepatic clearance of indocyanine green, thereby increasing PDR [3, 4, 6]. In parallel, rapid and intense tissue fluorescence uptake during ICGFA is an intuitive surrogate of adequate perfusion, whereas the development of a delayed or attenuated signal likely reflects impaired inflow and reduced clearance capacity. Post hoc analysis of ICG videos here showed that a shorter time to reach maximum intensity in well-perfused regions may be associated with elevated PDR. Moreover, the observed association between computational metrics from both assessment methods (PPS and ICGFA) suggests a degree of concordance between these approaches meaning that analytical evaluation of ICGFA may augment visual interpretation and add useful granularity by providing quantitative descriptors to the intraoperative assessment of perfusion.

This study is naturally limited by being small. In addition, the patients studied are heterogeneous with regard to disease and operation nature, and only a small percentage had major complications. Results of inferential tests should therefore be interpreted with caution, and effect sizes and descriptive statistics are provided to support such interpretation. Additionally some timepoint data was lost due to the delicate nature of the PPS test, especially intraoperatively, likely due to detachment of the finger probe under the drape or coldness of the extremity affecting its read after commencement. The work was also impacted by the LiMON technology only providing a single value at conclusion when a minimum quality of read is met rather than a continuous time-series curve which would have allowed for richer data for comparisons. The underlying algorithm is proprietary precluding further methodological detail however the system is considered robustly valid when a reading is outputted encouraging confidence in the readings when generated. Although we acknowledge that measuring ICG clearance through PPS is imperfect, and that more accurate techniques are available [3], the choice of LiMON technology was justified by the avoidance of unnecessary clinical risk for the purpose of this exploratory study. Different ICGFA camera systems detect and display the NIR signal differently [39]. Computational analysis was also only feasible in a subgroup so providing only preliminary insights into the potential value of this approach.

## Conclusion

In summary, this pilot study highlights the feasibility of perioperative splanchnic perfusion characterisation through peripheral ICG spectrophotometry in conjunction with intraoperative ICGFA in colorectal surgery. Although limited by sample size, the observed associations highlight the potential of integrating quantitative ICG-based monitoring into clinical practice. Larger, standardised studies are warranted to validate these findings and to determine whether such measurements can meaningfully aid postoperative outcome prediction.

## Supplementary Information

Below is the link to the electronic supplementary material.

Supplementary file1 (PDF 128 KB)

Supplementary file2 (PDF 124 KB)
