# Supplementary material for: Perioperative splanchnic perfusion variation around colorectal surgery using both indocyanine green spectrophotometry and fluorescence angiography
Source: Surg Endosc. 2026 Mar 2;40(5):3987–95. doi: 10.1007/s00464-026-12680-1 (PMC13161004; doi:10.1007/s00464-026-12680-1)
Supplement: Supplementary file 1 — Supplementary file1 (PDF 128 KB) [file 464_2026_12680_MOESM1_ESM.pdf]

Supplementary Table 1: Median (IQR) values of PDR and ICGR15 at each measurement time point by aggregated cohort operative subtype. Between-group comparisons were performed using the Mann–Whitney U test.

			Proximal resections (N=9) [#]	Distal resections (N=11)*	P value
PDR (%/min)	Preoperative (T1)		**20.5** (19.9 – 32.5)	**23.9** (20.0 – 29.2)	0.730
	Intraoperative (T2)		20.6 (18.6 – 25.6)	**17.9** (17.1 – 20.3)	0.210
	Postoperative (T3)		**20.6** (18.8 – 25.5)	**23.1** (21.6 – 26.0)	0.310
	Δ Pre-Intra		**-3.0** (-6.0 – -0.8)	**-1.4** (-11.2 – 2.7)	0.902
		%	**-14.8%** (-25.3 – -2.6)	**-7.2%** (-30.9 – 15.0)	0.710
	Δ Pre-Post		**-4.0** (-8.6 – 0.4)	**-1.7** (-9.7 – 0.5)	0.885
		%	**-13.7%** (-29.7 – 2.0)	**-7.2%** (-33.9 – 2.3)	0.888
	Δ Intra-Post		**4.6** (2.8 – 8.5)	**-1.3** (-4.3 – 1.0)	0.088
		%	**28.4%** (13.8 – 42.7)	**-6.1%** (-13.2 – 6.0)	0.145
ICGR15 (%)	Preoperative (T1)		**4.6** (0.8 - 5.1)	**2.8** (1.3 - 5.0)	0.757
	Intraoperative (T2)		**4.6** (2.4 - 6.2)	**6.8** (4.8 - 7.7)	0.210
	Postoperative (T3)		**4.6** (2.3 - 6.0)	**3.1** (2.0 - 3.9)	0.302
	Δ Pre-Intra		**2.7** (0.2 – 4.4)	**0.4** (-2.2 – 3.0)	0.456
		%	**56.3%** (14.4 – 148.2)	**22.2%** (-32.5 – 506.2)	0.902
	Δ Pre-Post		**1.0** (-0.3 – 1.7)	**1.0** (-0.5 – 2.1)	0.958
		%	**76.9%** (-6.2 – 262.0)	**36.2%** (-9.3 – 519.6)	0.758
	Δ Intra-Post		**-3.0** (-4.4 – -1.7)	**0.6** (-1.0 – 1.1)	0.140
		%	**-50.0%** (-72.1 – -35.4)	**21.3%** (-13.5 – 97.1)	0.088

[*] Included in this group are left hemicolectomies, anterior resections, and abdominoperineal resections. Missing values: n=2 at T1, n=2 at T2, n=0 at T3.
[#] Included in this group are right hemicolectomies and ileocaecal resections. Missing values: n=0 at T1, n=2 at T2, n=1 at T3.
