# Supplementary material for: Perioperative splanchnic perfusion variation around colorectal surgery using both indocyanine green spectrophotometry and fluorescence angiography
Source: Surg Endosc. 2026 Mar 2;40(5):3987–95. doi: 10.1007/s00464-026-12680-1 (PMC13161004; doi:10.1007/s00464-026-12680-1)
Supplement: Supplementary file 2 — Supplementary file2 (PDF 124 KB) [file 464_2026_12680_MOESM2_ESM.pdf]

**Supplementary Table 2.** Median (IQR) values of PDR and ICGR15 at each measurement time point by aggregated postoperative outcome cohort. Between-group comparisons were performed using the Mann–Whitney U test.

| | | | No postoperative complications (N=16) * | Any Postoperative complications (N=12) # | P value |
|---|---|---|---|---|---|
| PDR (%/min) | **Preoperative (T1)** | | **24.9** (19.5 - 30.5) | **20.4** (18.5 – 27.5) | 0.446 |
| | **Intraoperative (T2)** | | **17.7** (15.2 - 21.7) | **20.6** (17.1 – 25.5) | 0.413 |
| | **Postoperative (T3)** | | **24.1** (20.3 - 26.8) | **22.5** (18.4 – 29.9) | 1.000 |
| | **Δ Pre-Intra** | | **-3.0** (-6.8 – 0.4) | **1.3** (-7.3 – 5.2) | 0.272 |
| | | % | **-14.8%** (-30.3 – 1.6) | **6.4%** (-17.7 – 26.9) | 0.065 |
| | **Δ Pre-Post** | | **-4.0** (-9.4 – 0.4) | **-1.3** (-5.2 – 5.6) | 0.219 |
| | | % | **-14.2%** (-33.4 – 1.6) | **-4.2%** (-21.3 – 32.3) | 0.263 |
| | **Δ Intra-Post** | | **2.8** (-1.7 – 7.9) | **4.0** (-1.7 – 10.9) | 1.000 |
| | | % | **13.8%** (-5.4 – 39.3) | **32.7%** (-8.3 – 47.6) | 0.899 |
| ICGR15 (%) | **Preoperative (T1)** | | **2.4** (1.0 – 5.4) | **4.7** (1.9 – 6.4) | 0.446 |
| | **Intraoperative (T2)** | | **7.1** (3.9 – 10.2) | **4.6** (2.2 – 7.7) | 0.413 |
| | **Postoperative (T3)** | | **2.7** (1.8 – 4.8) | **3.4** (1.1 – 6.3) | 0.936 |
| | **Δ Pre-Intra** | | **2.7** (-0.2 – 5.7) | **-1.0** (-3.1 – 0.9) | **0.041** |
| | | % | **56.3%** (-8.3 – 182.4) | **-17.2%** (-52.9 – 309.8) | 0.272 |
| | **Δ Pre-Post** | | **1.2** (-0.3 – 3.2) | **0.2** (-3.3. – 1.4) | 0.219 |
| | | % | **79.6%** (-6.1 – 320.5) | **22.2%** (-49.7 – 137.7) | 0.219 |
| | **Δ Intra-Post** | | **-1.7** (-3.7 – 0.3) | **-1.8** (-5.4 – 0.6) | 0.766 |
| | | % | **-35.4%** (-65.3 – 33.3) | **-57.1%** (-81.8 – 28.3) | 1.000 |

* Missing values: n=1 at T1, n=4 at T2, n=3 at T3.

# Missing values: n=3 at T1, n=1 at T2, n=0 at T3
